# The Parallel Analysis of Phase Sensitive Inversion Recovery (PSIR) and Double Inversion Recovery (DIR) Images Significantly Improves the Detection of Cortical Lesions in Multiple Sclerosis (MS) since Clinical Onset

**DOI:** 10.1371/journal.pone.0127805

**Published:** 2015-05-26

**Authors:** Alice Favaretto, Davide Poggiali, Andrea Lazzarotto, Giuseppe Rolma, Francesco Causin, Paolo Gallo

**Affiliations:** 1 The Multiple Sclerosis Centre - Veneto Region (CeSMuV), Department of Neurosciences SNPSRR, Padova, Italy; 2 Neuroradiology Unit, University Hospital of Padova, via Giustiniani 5, Padova, Italy; University of Düsseldorf, GERMANY

## Abstract

**Background:**

Double inversion recovery (DIR) detects only a minority (<20%) of cortical lesions (CL) in multiple sclerosis (MS). Phase-sensitive inversion recovery (PSIR) was suggested to be substantially superior to DIR in the detection of cortical lesions (CL). These two sequences might be complementary.

**Objectives:**

To analyze CL frequency and type in MS patients having different disease duration and disability, including patients at clinical onset, and to discern more correctly the artifacts, by combining DIR and PSIR images.

**Patients and Methods:**

40 patients were enrolled in the study: 10 clinically isolated syndrome/early relapsing remitting MS (CIS/eRRMS), 24 relapsing remitting MS (RRMS), 6 secondary progressive MS (SPMS). DIR and PSIR images were jointly used to classify lesions as purely intracortical (IC), leukocortical (LC) and juxtacortical (JC).

**Results:**

PSIR disclosed CL in 100% of the patients and was capable of identifying more than four times lesions (455.5%, p<0.00001), especially IC (mean numbers: 36.5 in CIS/eRRMS, 45.0 in RRMS and 52.3 in SPMS) and LC (mean numbers: 10.9 in CIS/eRRMS, 20.1 in RRMS and 25.3 in SPMS), compared to DIR (p<0.00001). CL number was significantly higher in SPMS compared to RRMS (p<0.0001). Artifacts were more accurately identified by comparing the two sequences.

**Conclusions:**

Our study confirms the higher ability of PSIR in disclosing and classifying CL. The presence of CL in all CIS patients further points out the relevance of cortical pathology in MS. Whether the parallel analysis of DIR and PSIR images may be useful for diagnostic purposes, especially when a diagnosis of MS is suspected but not confirmed by routine MRI, needs to be evaluated in larger patient series. The analysis of the cortex by DIR and PSIR may also allow a better stratification of the patients for prognostic and counseling purposes, as well as for their inclusion in clinical studies.

## Introduction

Increasing evidence indicates that cortical pathology plays a major role in determining the progressive accumulation of physical and cognitive disability observed in multiple sclerosis (MS) patients [1–4]. Over the last decade, both focal inflammation and diffuse atrophy have been largely investigated in MS by means of several magnetic resonance imaging (MRI) methodologies. Among these, the double inversion recovery (DIR) sequence has been used to demonstrate cortical inflammatory lesions (CL) [5–8] and, although a minority (<20%) of histologically demonstrated CL can be detected by this sequence [9–11], they were found to increase the specificity of the MRI diagnostic criteria for MS [12] and to associate with physical and disability [1,3,13,14].

Considering the heterogeneity of MS clinical course, the accurate identification and localization of CL, especially during the early disease phases, might be important for understanding their clinical (diagnostic and prognostic) significance, and for a better characterization of the pathological process that occurs in MS, especially the relationship existing between cortical inflammation, neurodegeneration and disability. However, even when high-resolution DIR sequence is applied [3,5,8,13,14], two main pitfalls of DIR cannot be avoided. First, it does not always allow a correct identification of the two main CL subtypes recognized histologically, i.e., pure intracortical (IC) and leukocortical (LC, mixed white/grey matter lesions) [15]. In addition, the differentiation of LC lesions from juxtacortical (JC, confined in the subcortical white matter) lesions is challenging and sometimes impossible. Second, its intrinsic signal-to-noise ratio does not permit to increase the resolution we have at present obtained (thus missing the identification of small oval IC lesions) while maintaining a time of acquisition applicable for clinical purposes. Thus, to what extent our interpretation of DIR images is accurate and reproducible is unclear.

Recently, preliminary reports have suggested that phase sensitive inversion recovery (PSIR), a MRI sequence that associates a significantly higher resolution (improved grey/white contrast and better signal/noise ratio) with a clinically acceptable time of acquisition, is capable to detect about three folds more CL than DIR and to allow a better separation of LC lesions from the JC ones [16–19].

On the base of the few data available in the literature, we tested whether the parallel analysis of DIR and PSIR images could allow not only a more accurate (number and type) identification of CL, but also a more correct re-evaluation of the subtle signal changes observed on DIR images that, in a very restricted way of analysis and in line with the currently available guidelines, are not interpreted as lesions. To this aim, we obtained DIR and PSIR images with a 3T MRI from a cohort of 40 patients having wide disease duration and disability score, including clinically isolated syndromes [20] (CIS) and early relapsing remitting [21] MS patients (eRRMS, disease duration <3years). The parallel analysis of DIR and PSIR disclosed an impressive high number of signal abnormalities that could be attributed to CL, further remarking the relevance of cortical pathology in MS. Moreover, a better identification and interpretation of the artifacts observed with each sequence could be also achieved.

## Materials and Methods

### Patients

Forty patients (Table 1) were enrolled in the study. Ten patients (7F/3M) were clinically isolated syndromes [20] suggestive of MS (CIS, 7 patients) or very early relapsing remitting MS (eRRMS, 3 patients) [21] with a very short disease duration (0.9±0.6 years), dissemination in space of lesions at MRI and the presence of IgG Oligoclonal bands in the cerebrospinal fluid (CSF). Twenty-four (13F/11M) were relapsing-remitting MS (RRMS) with a mean disease duration of 9.6±7.2 years; 6 (5F/1M) had secondary progressive MS (SPMS) and a disease duration of 18.6±10.6 years [22]. In order to obtain a comprehensive view of the focal cortical pathology in different disease stages, the patients were selected to comprise a wide range of disease duration (0.33–41.5 years) and disability (Expanded Disability Status Scale, EDSS, range: 1–7.5). The study was approved by the local Ethics Committee (Comitato Etico per la Sperimentazione, Azienda Ospedaliera—Università degli Studi di Padova). All patients gave written informed consent.

**Table 1:** Demographic and clinical characteristics of the patients included in the study.

	F/M	Age	Disease Duration	EDSS
**CIS/eRRMS (10)**	7/3	30.0±7.2	0.9±0.6	1.6±0.5
		[17–41]	[0.33–2.4]	[1–3]
**RRMS (24)**	13/11	40.9±7.2	9.6±7.0	2.5±1.4
		[25–53]	[1–24.5]	[1–6.5]
**SPMS (6)**	5/1	44.5±9.8	20.2±10.9	6.6±0.5
		[27–56]	[8.7–41.5]	[6–7.5]

Data are expressed as mean ± SD and range (into brackets). Age and disease duration are expressed in years. EDSS = expanded disability status scale.

### Image Acquisition

3DT1, T2, FLAIR, DIR and PSIR images were acquired on a 3T Achieva TX system (Philips healthcare, Best, The Netherlands) with a 64-channel coil. The acquisition parameters for DIR and PSIR were the following: DIR = resolution 1x1x3mm, FOV 230x200mm, TR 13000ms, TE 10ms, TI 3400/325ms, Slices n40, time 3.5mins; PSIR: resolution 1x1x3mm, FOV 230x200mm, TR 7000ms, TE 13ms, TI 400ms, Slices n40, time 7mins.

### Image Analysis

Cortical lesions were identified on DIR scans using published consensus recommendations [8] and on PSIR scans following the rules previously published by others [16–17]. The identification and classification of CL was first done on DIR images (DIR-before PSIR, DIRb, i.e., blinded to PSIR) and after on PSIR (blinded to DIR) by three independent examiners (AF, DP, PG). Then, on the base of the consensus obtained on PSIR scans, DIR images were re-assessed in parallel with PSIR (lesion-by-lesion comparison) and all the faint shades of grey observed in the cortex in the previous analysis of DIR, but not considered lesions, were critically re-analyzed (DIRafter or DIRa). Lesion re-classification on both DIR and PSIR were scored by the three examiners independently, and changes in classification from either IC/LC/JC on DIR to IC/LC/JC on PSIR were obtained by consensus. All the work was supervised by two experienced neuroradiologists (FC, GR).

All possible sources of artifacts were analyzed. Enlarged Virchow-Robin spaces (VRS) can be observed on DIR, as a diffuse hyper-intense signal, that often does not allow the clear identification of single VRS, and on PSIR as a cribrous aspect of the tissue, particularly in the temporal cortex and insula. Since VRS can be mistaken for CL, a particular care was devoted to differentiate small CL from VRS. For these reasons the count of VRS was not included in the present study. Moreover, when a lesion was identified, the adjacent slices were carefully analyzed, in order to exclude both cortical vessels (particularly those that do not chase up the course of the cortical ribbon) and partial volume effects due to sulcal CSF.

### Statistics

Lesion distribution was tested for normality using the Kolmogorov-Smirnov test. As the distribution of lesions was found to be normal, parametric tests were used for further analysis. The t-Student test was used to compare PSIR and DIR lesion counts. When each clinical subtype was analyzed, Wilcoxon signed-rank test was used to compare PSIR and DIR lesion counts. Mann-Whitney U Test was used to compare PSIR lesion counts between different clinical subtypes. EDSS scores were compared by using Mann-Whitney U Test. SPSS Statistics 20 was used to perform the statistical analysis, and p<0.05 was considered significant.

## Results

The demographic and clinical features of the patient groups enclosed in the study are summarized in Table 1. Patients with CIS and eRRMS were pooled together given the very short disease duration (0.9±0.6 years; range 0.25–1.5). Differences in mean age, disease duration and in median EDSS score among the three groups were significant (p<0.001 for all parameters) as expected on the base of the clinical MS forms.

The mean number of the three lesion types (IC, LC, JC) scored on DIR images inspected before PSIR were slightly higher compared to those published in previous studies by others and us, and increased with disease duration (i.e., IC = from 5.1 in CIS to 13.8 in SPMS, p<0.0001). An impressive higher number of signal abnormalities that were recognized as lesions could be identified on PSIR images. In the whole MS cohort, PSIR was capable of identifying more than four times lesions than DIR (455.5%, p<0.00001), especially IC (mean numbers: 36.5 in CIS/eRRMS, 45.0 in RRMS and 52.3 in SPMS) and LC (mean numbers: 10.9 in CIS/eRRMS, 20.1 in RRMS and 25.3 in SPMS).

On the contrary, only a small number (about 2%) of lesions detected in DIR could not be confirmed on PSIR. As expected, SPMS showed the higher lesion numbers for all types, followed by RRMS and CIS/eRRMS patients (Table 1), and this was observed on both DIR and PSIR scans (p<0.001). In some SPMS patients, up to 15 lesions could be identified in a single brain slice and a mean of 101 IC+LC lesions per patient (range 51–166) could be scored in the entire brain by PSIR.

**Fig 1:**
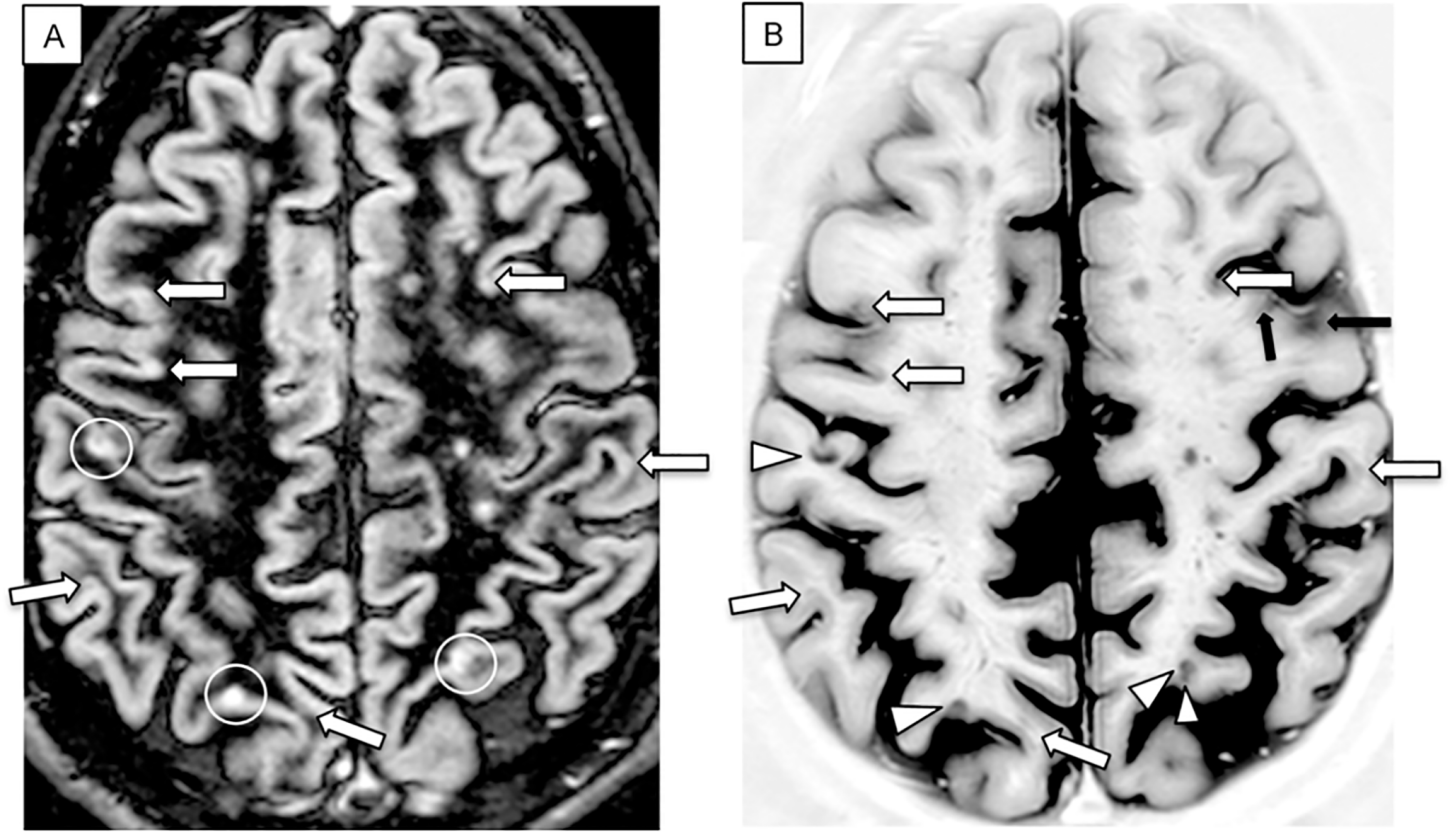
Axial DIR (A) and PSIR (B) images of a RRMS patient. Two LC and one JC lesions were identified by consensus on DIR (white circles) and confirmed by PSIR (arrow head). PSIR disclosed six more lesions (white arrows) that were not identified on DIR prior to PSIR. Black arrows indicate artifacts due to the partial visualization of the sulci.

When DIR and PSIR images were analyzed in parallel, on the base of PSIR images and given the similar morphology and the topographic correspondence, several faint changes in the hyper-intense signal of the cortical ribbon observed on DIR could be revised and definitely interpreted as CL by consensus (Fig 1). Nevertheless, the number of lesions detected by PSIR was still significantly higher (p<0.0001) when compared to that observed on DIRa.

**Fig 2:**
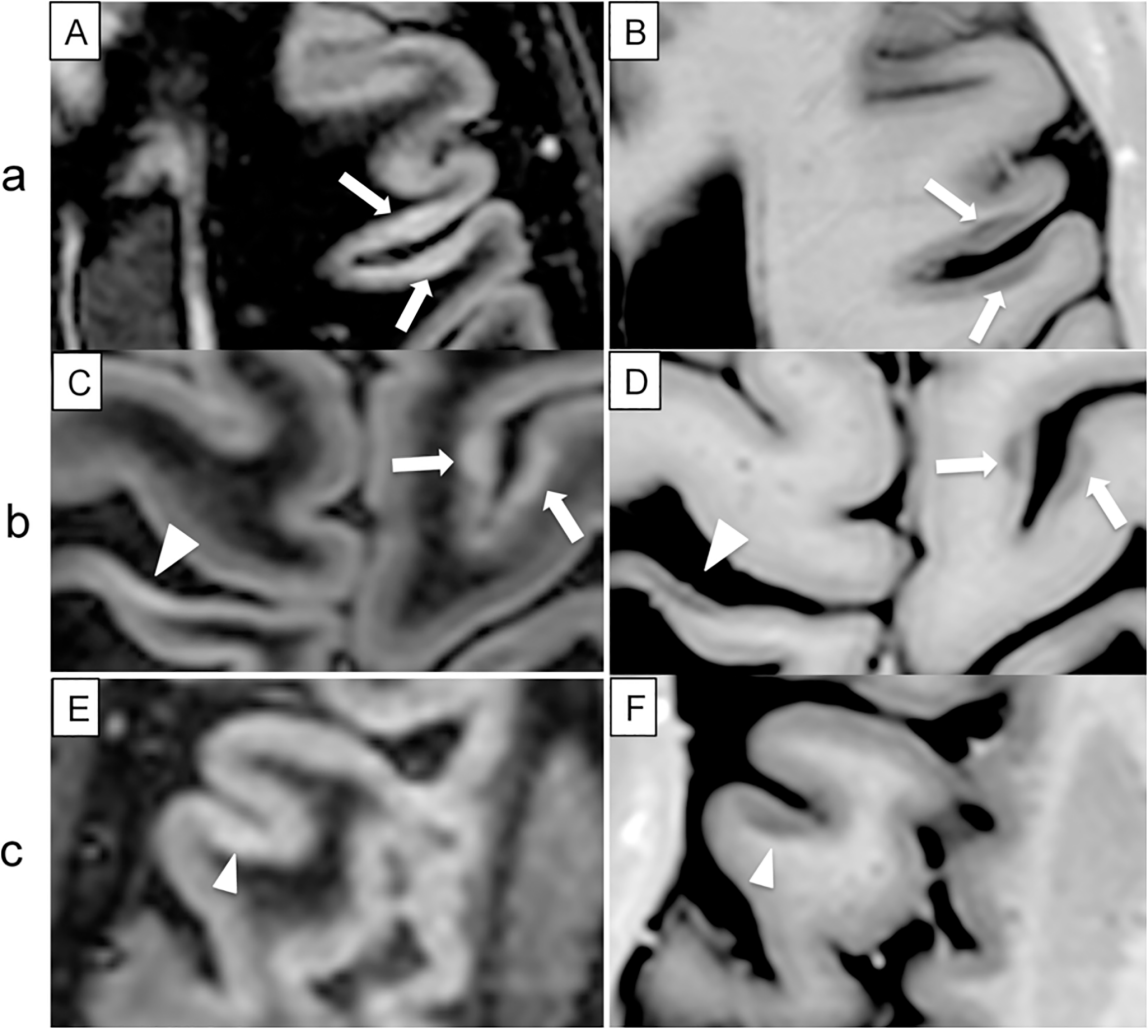
Images of CL from DIR scans (A, C, E) and PSIR scans (B, D, F) from three different patients. (a), two IC lesions (arrows) are observed on DIR (A) and confirmed on PSIR (B) images. (b), two IC lesions (arrows) are detected on DIR (C) and confirmed on PSIR (D) images. However, an additional ‘linear’ IC lesion can be observed on PSIR (arrow head); this lesion was not identified at the first analysis of DIR scan, but its signal intensity was re-evaluated as an IC lesion after PSIR analysis. (c), a LC (mixed) lesion is observed on PSIR (F), but not on DIR scan given the weak signal.

**Fig 3:**
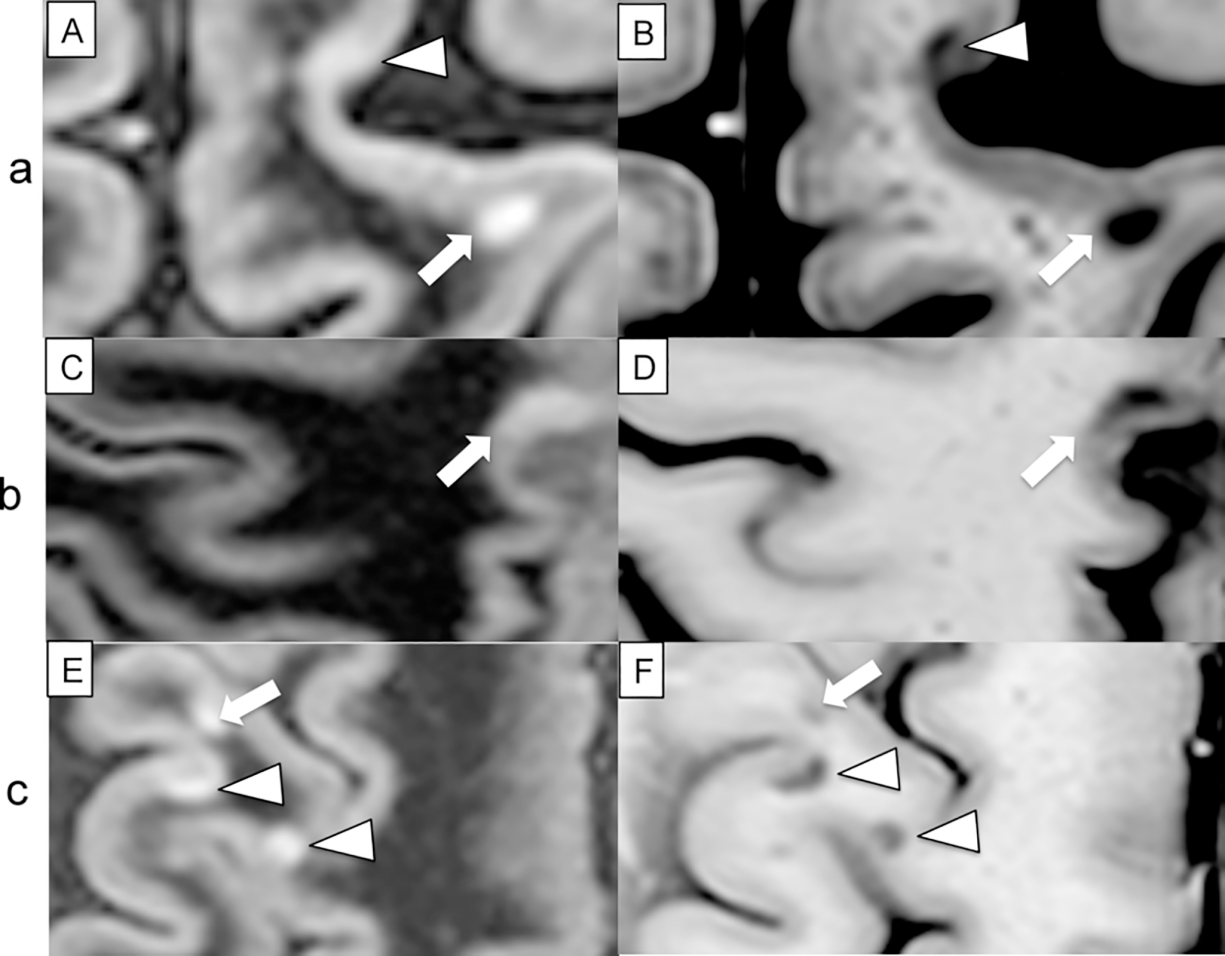
Images of CL from DIR scans (A, C, E) and PSIR scans (B, D, F) from three different patients. (a), a juxtacortical lesion (arrow) is observed on both DIR (A) and PSIR (B) images, but a leukocortical lesion (arrow-head) visible on PSIR was not confirmed on DIR images both before and after PSIR analysis. (b), a lesion that was classified as intracortical on DIR scans (arrow)(C), was re-classified as mixed on PSIR images (D). (c), two lesions classified as mixed (arrows-head) on DIR (E) were re-classified as juxtacortical on PSIR (F). A further juxtacortical lesion was observed on DIR (arrow) and confirmed on PSIR.

**Fig 4:**
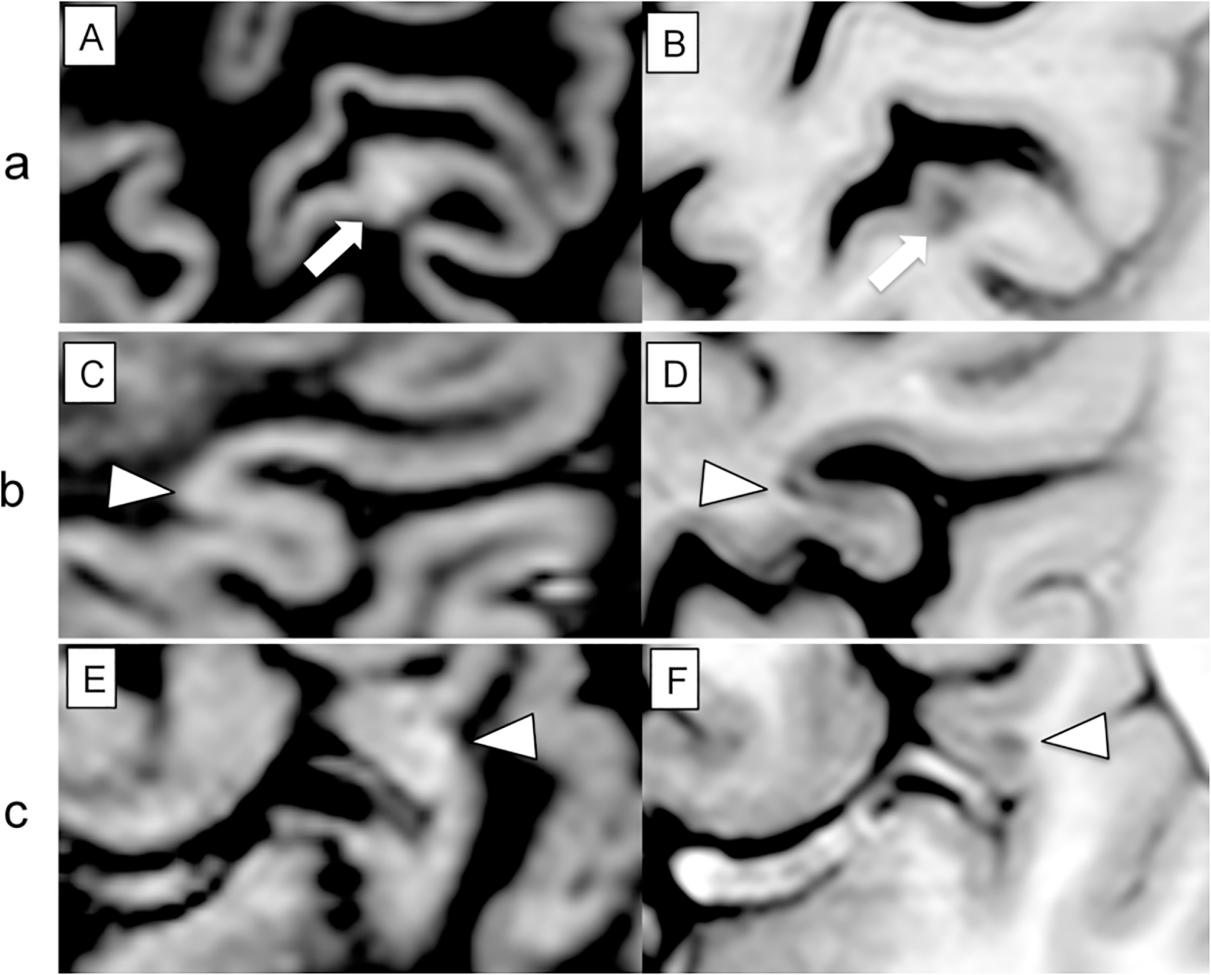
Images of CL from DIR scans (A, C, E) and PSIR scans (B, D, F) from three different patients. (a), the altered cortical signal (arrow) observed on DIR (A) could not be classified as a specific type of lesion, but was definitely considered a juxtacortical lesion on PSIR scan (B). (b), a small oval intracortical lesion (arrow-head) was observed at the bottom of the sulcus on PSIR images (D), but not on DIR images (C). (c), a small ovoid juxtacortical lesion (arrow-head) was identified on PSIR image (F), but was erroneously considered an artifact on DIR image (E).

Our findings confirm that PSIR allows the identification of more CL and their better classification as shown in Figs 2–4. IC lesions were seen in 40/40 patients with PSIR and in 34/40 patients on DIRb, ≥1 LC lesions were seen in 37/40 on PSIR (in 30/40 on DIRb), and ≥1 JC lesions were seen in 34/40 on PSIR (in 28/40 on DIRb). In agreement with literature data [16], PSIR allowed a more correct discrimination of LC and JC lesions compared to DIR. Indeed, more than 15% of the lesions observed on DIR were re-classified, especially the JC ones, and a better identification of CL morphology was obtained. Finally, we observed that a considerable number of wedge-shaped and small oval lesions were located at the deep infoldings of the cerebral sulci (Figs 2c and 3c), a finding that was particularly evident when DIR and PSIR were observed in parallel.

A non-significant trend was observed between cortical lesion number and EDSS score.

Artifacts are frequently observed on both DIR and PSIR scans and their differentiation from true lesions is often difficult. Thus, careful attention was devoted to exclude the artifacts that are commonly observed in ‘high-risk regions’ such as the anterior temporal lobes and insula. These areas, that may appear diffusely hyper-intense on DIR, mimicking ‘confluent lesions’, are shown by PSIR to be rich of enlarged VRS, forming a kind of ‘cribrous tissue’, especially in SPMS or RRMS with longer disease duration. Another source of artifacts are cortical vessels, and the parallel analysis of DIR and PSIR images significantly helped in their identification. The longitudinal sections of the vessels can be recognized by their hyper-intense signal on both DIR and PSIR scans. However, the cross-sectional images of the vessels might be slightly hyper-intense or iso-intense with the cortex on DIR and hypo-intense on PSIR. Therefore, slices contiguous to those with a suspected lesion were carefully analysed both on DIR and PSIR to exclude that cortical vessels would be falsely counted as lesions. We also observed that other sites of frequent artifacts, due to the signal noise produced by vessels, are the medial frontal lobes. Finally, due to the higher propensity of DIR to produce artifacts caused by vessels and CSF pulsation in the posterior fossa, images of infratentorial brain were not included in the present study.

## Discussion

On the base of the few preliminary reports available in the literature [16–19] that have already demonstrated the superiority of PSIR over DIR in the detection and classification of CL, our work was aimed to 1) analyze number and location of CL by combining the information obtained by the parallel analysis of DIR and PSIR images, 2) verify whether the interpretation of DIR images currently adopted is correct, 3) have more confidence in recognizing signal changes produced by artifacts on both sequences. To this aims, MS patients with different clinical forms of the disease, and a wide range of disease duration and EDSS score, were enrolled in the study. Since us and others have previously reported that the observation of CL in healthy individuals is an extremely rare event [3,6,12,16], the lack of a group of healthy controls cannot be considered a limitation of the study.

The first relevant finding is that CL could be detected by PSIR in all the patients, including CIS suggestive of MS and eRRMS. The high number of lesions detected by PSIR in these patients, although surprising and unexpected, is in line with histological studies [23,24] and with the observations that some MS may present with CL as they earliest pathologic event [25, 26], and indicates that this sequence gives a more realistic view of cortical pathology in MS compared to DIR. On the other hand, it has to be pointed out that the currently available PSIR sequence needs a 3mm thick slice for a good contrast-to-noise-ratio. Hence it does not allow a precise estimation of the lesion volume.

The better contrast and the lower signal-to-noise ratio of PSIR may partly explain the higher number of CL observed by this sequence compared to DIR. However, the magnetic field has also to be considered. Indeed, a comparative study demonstrated that the number of the DIR-detectable CL increases with the power of the magnetic field used, i.e., 3T vs 1.5T [14]. This is in line with our experience: the CL number observed by DIR in our previous studies using a 1.5T scanner was lower compared to that scored in the present work. It has also to be noted that the use of ultrahigh-field MRI (7T) was found to improve the detection of CL by FLAIR [27], a sequence that it’s recognized to be unsuitable for this purpose.

The identification of several IC lesions in all CIS/eRRMS indicates that could help in seeking evidence for cortical lesions at clinical onset in cases of suspected MS. Moreover, this finding indicates that cortical inflammation goes along with white matter inflammation since the beginning of the disease. However, despite the significant increase in CL number observed in SPMS compared to both CIS/eRRMS and RRMS, and the significant difference in EDSS score between the patient groups, no significant correlation could be demonstrated between CL number and EDSS, thus indicating that other aspects of grey matter pathology (i.e. diffuse atrophy) probably play a more relevant role in determining the progressive increase of physical and cognitive disability observed in MS.

Faint changes in the cortical hyper-intense signal may be observed in MS cortex on DIR sequence and, applying a restrictive interpretation, they are usually not considered CL. Our study shows that in many cases, on the base of the parallel evaluation of PSIR and DIR images, these changes can be re-considered true lesions. However, despite the re-analysis of DIR sequence after PSIR, the number of CL observed on PSIR was significantly higher compared to that scored on DIRa, further strengthening literature data [16–19]. Our data also confirm the higher resolution of PSIR in differentiating IC from LC and LC from JC lesions, thus allowing a more precise definition of the CL load that might be important in studies on cognitive decline and physical disability.

From an immune-pathological point of view, particularly interesting is the high number of curvilinear or oval lesions observed by PSIR at the deep infolding of the cerebral sulci. Indeed, meningeal B-cell follicle-like aggregates have been predominantly found in the shielded confines of the deep cerebral sulci, and only rarely at the crown of the gyri [28,29]. The presence of lymphoid aggregates has been interpreted as expression of a state of elevated meningeal inflammation that was suggested to play a role in causing cortical demyelination. Given the dynamic of the CSF flow, it might be possible that the deep cortical sulcus provides the optimal biological and physical condition for meningeal inflammation and cortical demyelination.

From a methodological point of view, the parallel analysis of DIR and PSIR images, as done in the present work, might significantly improve CL identification and classification, since the distinction of CL from non-pathological areas of altered signal intensity can be very difficult with either DIR or PSIR alone. Indeed, several ambiguous CL could be more clearly identified observing DIR and PSIR images in parallel. Moreover, the identification of small lesions at the bottom of the cerebral sulcus on DIR was clearly facilitated by the parallel observation of the corresponding PSIR image. We confirm that Virchow-Robin spaces can be identified on PSIR and are especially gathered in temporal cortex and insula. In our experience, the review of FLAIR and T2 scans in conjunction with the PSIR scan only rarely helps to detect cortical CL or to better characterize artifacts. We found generally good inter-rater and intra-rater consensus for the identification of cortical lesions, although inter-rater classification of LC and JC lesions, in agreement with previous reports [16,17] was less consistent.

In conclusion, the parallel analysis of DIR and PSIR images discloses an extraordinary high number of CL in MS, even at clinical onset and at the very early stages of the disease, thus putting the neuroimaging findings a little closer to the histo-pathological ones. Moreover, the two sequences together significantly help the examiner in the correct identification of the lesion types and artifacts. Indeed, it’s now possible to observe more accurately the extent and the severity of cortical inflammation in vivo and to distinguish it from subcortical white matter inflammation, with possible important diagnostic and prognostic consequences. Since the neuroimaging of CL is an evolving field of research and may have important clinical rebounds a consensus on well-defined criteria for a more robust comparison between DIR and PSIR, especially for a correct identification of lesion subtypes, is needed.
